# Work autonomy and its associated factors among health professionals in public hospitals of North East Ethiopia

**DOI:** 10.1038/s41598-024-66865-6

**Published:** 2024-07-08

**Authors:** Ali Yimer, Amare Zewdie, Amsalu Feleke, Endalkachew Dellie, Mohammed Ahmed, Seada Seid, Wubshet Debebe, Hassen Ahmed, Wolyu Korma, Mohammed Adem, Abdulaziz Kebede

**Affiliations:** 1 Department of Public Health, College of Health Sciences, Woldia University, Woldia, Ethiopia; 2 Department of Public Health, College of Medicine and Health Sciences, Wolkite University, Wolkite, Ethiopia; 3 Department of Health Systems and Policy, Institute of Public Health, College of Medicine and Health Sciences, University of Gondar, Gondar, Ethiopia; 4 Department of Biomedical Science, School of Medicine, College of Health Sciences, Woldia University, Woldia, Ethiopia; 5 Department of Public Health, College of Medicine and Health Sciences, Werabe University, Werabe, Ethiopia; 6 Department of Public Health, College of Medicine and Health Sciences, Wollo University, Wollo, Ethiopia

**Keywords:** Work autonomy, Health professionals, Northeast, Ethiopia, Health care, Health occupations

## Abstract

A low level of work autonomy is the bottleneck for the health service delivery and the quality of the service. Although work autonomy is the pillar of organizational commitment and a means of employee retention mechanism, information about the magnitude of work autonomy among health professionals is limited in Ethiopia. Therefore, this study aimed to assess work autonomy and its predictors among health professionals working in public hospitals of Northeast Ethiopia. Institution-based cross-sectional study was conducted from March 24 to April 24, 2021, among health professionals using a stratified sampling technique. Variables with a p-value of < 0.25 in bivariable analysis were included in the multivariable analysis and variables with a p-value of < 0.05 in multivariable analysis were regarded as significantly associated factors. The overall good work autonomy in public hospitals (Dessie and Boru Meda Hospital) of North East Ethiopia was 54.5% (95% CI 54.48–54.53). Satisfaction with organizational policy and strategy (AOR 2.34, 95% CI 1.29–4.25), satisfaction with supervisor support (AOR 7.20, 95% CI 3.97–13.07), good health service delivery planning practice (AOR 1.88, 95%CI: 1.13–3.13), being married (AOR 4.26, 95%CI: 2.06–8.82) being pharmacy professionals (AOR 0.44, 95% CI 0.19–0.98), and being anesthesia and radiology professionals (AOR 4.66, 95% CI 1.65–13.19) were significantly associated with work autonomy of health professionals. More than half of the health professionals working in public hospitals in Northeast Ethiopia are autonomous in their work. Satisfaction with organizational policy and strategy, satisfaction with supervisor support, having good health service delivery planning practice, being married, and type of profession were significantly associated factors in public hospitals. Thus, strengthening strategies aimed at shaping poor health service delivery planning practices and dissatisfaction of employees concerning supervisor support and organizational policy might have a substantial contribution to improving the work autonomy of health professionals.

## Introduction

Work autonomy refers to a degree of independence and freedom employees are required to do their work. Specifically, it relates to the pace at which work is completed, its order of completion, and a person’s freedom to work without micromanagement^1^. Autonomy doesn’t mean giving people the freedom to choose how to do their best work without completely removing accountability or regulations and processes^2^. Those things can still be observed, and in fact should be observed, to create proper autonomy. It’s more about empowering the individual to do their best work. When we talk about an autonomous workplace, we aren’t talking about “let’s go break all the rules,” but rather “let’s be transparent about the constraints we have to work around”^2^. It seems perspicuous that a feeling of volition and freedom positively underpins creative thinking, and most people who ever tried to understand out of the box under pressure and fault-finding control^3,4^. Health system organizations hire tremendous health professionals, employees who are using a set of professional norms^5^. Due to the characteristics of these employees' work nature, most administrative officials realize using Mintzberg’s theory^6^, that professionals should have a great level of autonomy from supervision at the time of applying their professional skills^7^.

Improving health professionals' work autonomy promotes their knowledge and experience^8^. Moreover, a health professional's work autonomy plays a significant role in patients, health professionals, and health institutions in terms of motivation, turnover reduction, and retention mechanisms of employees^8–10^. The critical and relevant characteristic of work autonomy is that it is based on a specific body of knowledge and skills that is not mastered by people outside the profession. Due to this case, the demand for autonomy is highly probable to emanate from the situation of the work itself. Professionals may find the effects of their organizational goals concerning the restriction of their freedom to provide care for their patients in their way, skills, and Knowledge^11^. Work situations that consider autonomy a part of basic the psychological needs of human beings go hand in hand with workplace health and emotional well-being. Various studies showed the importance of autonomy for health, creativity, and overall well-being^12,13^. Theories have also shown the role of autonomy in intrinsic motivation and well-being^14^.

Low work autonomy in every organization particularly health system organizations is still an existing problem that is a critical barrier to health service delivery and service quality at large^8^. Various epidemiological studies found a link between increased risk of cardiovascular disease and high job strain^15,16^. Undermining work autonomy affects not only workplace health at the level of an individual but also the workplace as an organization^17^. Based on a survey conducted in 1999, the European Commission estimated the yearly cost of work-related stress at €20 billion a year^18^. The magnitude of work autonomy is not well addressed yet according to scientifically available data. However; a study conducted in Oromia regional state revealed a magnitude of good work autonomy 46.13%^19^. Poor work autonomy could lead to several consequences. The main destructive consequences of inadequate work autonomy are poor job satisfaction, sluggish work engagement, low job performance, employees’ high turnover, conflict between health professionals and their managers, poor health service quality, and inefficient workflow^20–24^.

There are different factors known to affect work autonomy. Among these factors sex, age, marital status, educational status, experience, job satisfaction, and salary are the most fundamental factors with a significant role in health professionals’ work autonomy^11,19,21,25,26^. Other variables such as work unit and type of profession have also been considered as associated factors^19,27^.

The ultimate goals of the WHO health system building blocks of health care are health improvement, responsiveness, and financial and social risk protection^28^. This stated goal will only be possible if there are autonomous and committed health professionals who can translate the national aspirations and desires of the community into a reality^28^. The vision of the newly revised 2017 Ethiopian Government Health Policy is to see a healthy, productive, and prosperous generation. However; this can be effectively achieved when there are autonomous, experienced, and inspired staff who are accessible and responsible for providing the health service needs of the community^29^.

Some studies have been done on work autonomy. However; most of the studies are not on health professionals’ work autonomy^30^. No study addresses the whole health professionals’ work autonomy, particularly in Ethiopia. Some important variables such as supervision support, health service delivery planning practice, co-worker relationship, and organizational policy and strategy have not been included in previous studies^8,19^. Therefore; this study aims to assess work autonomy among health professionals working in public hospitals in Dessie City and Boru Meda General hospitals, Northeast Ethiopia.

## Methods

### Study design and setting

An institutional-based cross-sectional study was conducted between March and April 2021 at public hospitals in Dessie City. Dessie City is located 401 km far from Addis Ababa, the capital city of Ethiopia, and 488 km from Bahir Dar, the capital city of Amhara National Regional State^31^. In the city, there are five general private hospitals, two public hospitals, eight public health centers, and ten health posts to serve Dessie City and the catchment areas of more than 8 million residents from east Amhara, part of Tigray and Afar Region^32^. Boru Meda hospital currently (2021) has 40 beds for leprosy and other dermatology cases, in addition to other case teams. It also has three dermatology outpatient clinics with two dermatologists: a tropical dermatology professional and a health officer who has dermatology and leprosy training.

### Populations

Those health professionals who served for a minimum of six months in the city’s public hospitals (Dessie Specialized Referral Hospital and Boru Meda General Hospital) were the population included in this study.

### Eligibility criteria

Inclusion Criteria: All health professionals who work in the city’s public hospitals (Dessie Specialized Referral Hospital and Boru Meda General Hospital) during the study period.

Exclusion Criteria: Health professionals who work for less than six month was excluded from the study.

### Sample size calculation

For the determination of work autonomy, the sample size was calculated based on the single population proportion formula. The calculation used a 46.13% proportion from a study conducted in Wollega^19^, a 5% margin of error, a 95% confidence level, and a 10% nonresponse rate with the following formula:

$${\text{n}} = \left( {{\text{z}}_{\alpha /2} } \right)^{2} \;{\text{p}}\;\left( {1 - {\text{p}}} \right)/{\text{d}}^{2} \;\;\;{\text{n}} = \left( {1.96} \right)^{2} (0.4613)\;\left( {0.5387} \right)/0.05^{2}$$

Z_α/2_ = confidence interval at 95%, n = 381.

d = level of significance at 5%

p = population proportion 46.13%

adding a 10% non-response rate, the total sample size was 419.

### Sampling procedures and techniques

A stratified sampling technique based on profession type was followed to select health professionals from seven departments. Health professional Payrolls of Dessie Specialized Referral Hospital and Boru Meda General Hospital from the Human Resource Management were used as a frame for each professional category. Next, the sample size was proportionally allocated to number of the health professionals for each hospital. Finally, the study participants were selected by using a simple random sampling technique.

### Study variables and their measurement

The dependent variable is work autonomy. Socio-demographic factors (age, sex, marital status, educational status, work experience, type of profession, monthly salary, living condition, and work unit), personal factors (manager’s attitude towards time management, health service delivery planning practice, and employees’ attitude towards time management) and organizational factors (retention and reward, co-worker relationship, organizational policy, and strategy and supervision support).

The dependent variable is the work autonomy of health professionals (Good/Poor) which describes the employees’ self-direction in initiating and continuing their work behaviors and making decisions. It was measured by four items having five Likert scales ranging from 1 strongly disagree to 5 strongly agree. It was categorized as good if the responses were greater than the mean value and poor if the responses scored equal to and below the mean value^33^.

*Manager’s attitude towards time management* indicates the managers’ outlook regarding time management. It was measured using five items having a five-point Likert scale. If a participant’s response scored above the mean value, it was represented as a good attitude if not a poor attitude^34^.

*Employees’ attitude towards time management* shows the likelihood of employees’ intention on effective time management utilization. It was measured by three items having a Likert scale out of five points. If the participant’s response score was above the mean value, it was represented as a good attitude if not a poor attitude^34^.

*Co-worker relationship* means the employee's interpersonal relationships with each other. This was measured by using 3 items each scored five-point Likert scale. It was categorized as good if the responses scored greater than the mean value and poor if the responses scored equal to and below the mean value^33^.

*Supervisor support* measured by six items having five Likert scales ranging from 1 strongly disagree to 5 strongly agree. It was categorized as good if the responses scored greater than the mean value and poor if the responses scored equal to and below the mean value^33^.

*Recognition and reward* describe the employees’ feelings towards the timeliness and fairness of recognition and reward. It was measured by using 4 items each scoring a 5-point Likert scale It was categorized as satisfied if the responses scored greater than the mean value and unsatisfied if the responses scored below and equal to the mean value^35^.

*Organizational policy and strategy* could be stated as the participants’ feelings on the application of organizational policies and strategies. It was measured by using 5 items each scored 5-point Likert scale. It was categorized as good if the responses scored greater than the mean value and poor if the responses scored equal to and below the mean value^35^.

*Health service delivery planning practice* describes setting goals, scheduling, and outlining tasks. It was measured by 4 items having five-point Likert scales. Those having above the mean value were regarded as having a good plan and those scoring below and equal to the mean value were represented as having a poor plan^35^.

### Data collection tools and procedures

A structured and pretested questionnaire was used to collect the data. The self-administered questionnaire which was adapted from different studies was used^19^. Five trained Diploma Nurses and three Bachelor of Science (BSc) nurses collected and supervised the data respectively. Cron-Bach’s Alpha was done to check the internal consistency of the tools and each item scored above 0.7.

### Data quality assurance

At Woldia General Hospital, 5% of the sample size was taken as a pretest to test the self-administered questionnaire before the actual data collection period. Amendments to the form, such as ambiguous terms and imprecise questions, were checked appropriately. The primary investigator provided a one-day training on the purpose of the study, the instrument, and the data collection procedures. Data collectors and supervisors were hired based on their research expertise. Experts in public health research also assessed the tool. The Principal Investigator and Supervisors reviewed each questionnaire once it was collected. Each data collector reviewed each participant's completed questionnaire daily to confirm the accuracy of the data. Every day, the Supervisors and Principal Investigator reviewed each questionnaire and verified its accuracy.

### Data processing and analysis

The accuracy and consistency of each piece of information were examined before being collected and coded. It was finally entered into EPI-DATA version 4.6. The entered data was exported to the statistical package for social sciences (SPSS version 25) (IBM, USA) software for analysis. Tables were utilized to present the data and frequencies and cross-tabulations were employed to summarize the descriptive statistics of the data. With 95% confidence intervals, crude and adjusted odds ratios were used to determine the significance of the association. A binary logistic regression model was used to study the initial bivariable correlations between each independent variable and outcome variable to determine the relationship between the various predictor factors and the dependent variable. To exclude potential confounding variables, a multivariable analysis was performed on the independent variables with a p-value < 0.05 with a 95% confidence interval were regarded as factors significantly associated with work autonomy.

### Ethical approval and consent to participate

Ethical clearance was obtained from the Institutional Review Board (IRB) of the University of Gondar with reference number IPH/1452/2013. A written permission letter was obtained from hospitals managers. Participants were formally informed about the purpose of the study. Confidentiality was maintained by omitting direct personal identifiers on the questionnaire, using code numbers, storing data locked with a password, and not misusing or wrongfully disclosing their information. Participants were also informed that participation was voluntary and they could withdraw from the study participation at any stage if they were not comfortable with the investigation. Written informed consent was obtained from the study participants before the study commencement.

## Results

A total of 409 participants responded to the self-administered questionnaire, resulting in a response rate of 97.6%. Among the participants, approximately 246 (60.1%) were male. The median age of the participants was 29 years, with an interquartile range of 27 to 33 years. Around 61.6% of the respondents were married and 71.6% of the participants resided with their families. The median monthly salary of study participants was 7071 (IQR: 6193, 9056) ETB. Furthermore, 50.6% of the participants were employed in inpatient work units within public hospitals (refer to Table 1).

**Table 1:** Socio-demographic characteristics of health professionals working in public hospitals in Northeast Ethiopia, 2021 (n = 409).

Variable	Category	Frequency (%)
Sex of participants	Male	246 (60.1)
	Female	163 (39.9)
Age category	20–24 years	43 (10.5)
	25–29 years	185 (45.2)
	30–34 years	101 (24.7)
	above 35 years	80 (19.6)
Marital status	Unmarried	157 (38.4)
	Married	252 (61.6)
Educational level	Diploma	67 (16.4)
	Degree and above	342 (83.6)
Work experience in years	0- 5 years	227 (55.5)
	6–10 years	138 (33.7)
	above 10 years	44 (10.8)
Living condition	With family	293 (71.6)
	Without family	116 (28.4)
Profession	Nurse	254 (62.1)
	Doctor	57 (13.9)
	Laboratory professionals	37 (9)
	Pharmacy	38 (9.3)
	Anesthesia and radiology	23 (5.6)
Monthly salary	below 6193 ETB	124 (30.3)
	6193–7071 ETB	165 (40.3)
	> 7071 ETB	120 (29.4)
Working unit	Outpatient	202 (49.4)
	Inpatient	207 (50.6)

*n* sample size, % percentage, *ETB* Ethiopian Birrr.

### Personal and organizational factors

Regarding personal and organizational factors, the majority of health professionals, specifically 363 individuals (88.8%), were satisfied with their co-worker relationships in public hospitals. However, a significant proportion of health professionals, particularly 57.5%, 66.3%, and 80.2% reported being dissatisfied with supervisor support, organizational policy and strategy, and recognition and reward systems respectively. Moreover, 42.1% of the respondents exhibited good practices in health service delivery planning (refer to Table 2).

**Table 2:** Personal and organizational factors among health professionals working in public hospitals of Northeast Ethiopia, 2021, (n = 409).

Variable	Category	Frequency (%)
Organizational policy and strategy	Unsatisfied	271 (66.3)
	Satisfied	138 (33.7)
Health service delivery planning practice	Poor	237 (57.9)
	Good	172 (42.1)
Employees’ attitude towards time management	Poor	352 (86.1)
	Good	57 (13.9)
Supervisor support	Unsatisfied	235 (57.5)
	Satisfied	174 (42.5)
Manager’s attitude towards time management	Poor	186 (45.5)
	Good	223 (54.5)
Recognition and reward	Unsatisfied	328 (80.2)
	Satisfied	81 (19.8)
Co-worker relationship	Unsatisfied	46 (11.2)
	Satisfied	363 (88.8)

*n* sample size,% = percentage.

### The magnitude of work autonomy

The overall good work autonomy in public hospitals was determined to be 54.5% (95% CI 54.48–54.53), indicating a generally positive perception of work autonomy among the health professionals working in the study area.

### Associated factors of work autonomy in public hospitals of Northeast Ethiopia

To identify factors associated with work autonomy in public hospitals in Northeast Ethiopia, logistic regression analysis was conducted using odds ratios (ORs) and 95% confidence intervals (CIs). Initially, bivariable logistic regression was performed to assess the association between each independent variable and the outcome variable. Thirteen variables with a p-value below 0.25 were selected as candidates for multivariable logistic regression analysis. Variables with a p-value less than 0.05 in the multivariable logistic regression analysis were considered statistically significant.

In the multivariable logistic regression analysis of public hospitals, five variables were found to be statistically significant. Health professionals who were satisfied with organizational policy and strategy had 2.34 times higher odds of having good work autonomy compared to those who were unsatisfied (AOR 2.34, 95% CI 1.29–4.25). Similarly, individuals who were satisfied with supervisor support had 7.20 times higher odds of having good work autonomy compared to their counterparts (AOR 7.20, 95% CI 3.97–13.07). Health professionals who demonstrated good health service delivery planning practices had 1.88 times higher odds of having good work autonomy (AOR 1.88, 95% CI 1.13–3.13). Furthermore, married health professionals were 4.26 times more likely to have good work autonomy compared to their unmarried counterparts (AOR 4.26, 95% CI 2.06–8.82). Additionally, anesthesia and radiology health professionals had 4.66 times higher odds of having good work autonomy compared to nurses (AOR 4.66, 95% CI 1.65–13.19). However, pharmacy health professionals had their work autonomy reduced by 56% compared to nurses (AOR 0.44, 95% CI 0.19–0.98) (refer to Table 3).

**Table 3:** Bivariable and multivariable logistic regression analysis of factors associated with work autonomy among health professionals working in public hospitals of Northeast Ethiopia, 2021(n = 409).

Variable	Category	Public hospitals (n = 409)			
		Work autonomy		COR (CI)	AOR (CI)
		Good	Poor		
Sex of participants	Male	142	104	1.38 (0.93–2.06)	1.02 (0.61–1.71)
	Female	81	82	1	1
Age category	20–24 years	26	17	1	1
	25–29 years	90	95	0.62 (0.32–1.22)	0.74 (0.28–1.95)
	30–34 years	56	45	0.81 (0.39–1.68)	0.67 (0.23–2.00)
	above 35 years	51	29	1.15 (0.54–2.47)	0.37 (0.12–1.16)
Educational level	Diploma	28	39	1	1
	Degree and above	195	147	1.85 (1.09–3.14)*	1.86 (0.94–3.69)
Marital status	Unmarried	72	85	1	1
	Married	151	101	1.77 (1.18–2.64) *	4.26 (2.06–8.82) **
Living condition	with family	166	127	1.35 (0.88–2.08)	0.70 (0.35–1.37)
	without family	57	59	1	1
Profession	Nurse	138	116	1	1
	Doctor	34	23	1.24 (0.69–2.23)	0.72 (0.33–1.54)
	Laboratory professionals	20	17	0.99 (0.50–1.98)	2.06 (0.86–4.93)
	Pharmacy	16	22	0.61 (0.31–1.22)	0.44 (0.19–0.98) *
	Anesthesia and Radiology	15	8	1.58 (0.65–3.85)	4.66 (1.65–13.19) *
Organizational policy and strategy	Unsatisfactory	119	152	1	1
	Satisfactory	104	34	3.91 (2.48–6.16) **	2.34 (1.29–4.25) *
Employees’ attitude towards time management	Poor attitude	59	76	1	1
	Good attitude	164	110	1.92 (1.27–2.92) *	1.57 (0.92–2.68)
Health service delivery planning practice	Having poor plan	76	103	1	1
	Having good plan	147	83	2.40 (1.61–3.58) **	1.88 (1.13–3.13) *
Supervisor support	Unsatisfactory	84	151	1	1
	Satisfactory	139	35	7.14 (4.52–11.27) **	7.20 (3.97–13.07) **
Recognition and reward	Unsatisfactory	164	164	1	1
	Satisfactory	59	22	2.68 (1.57–4.58) **	1.36 (0.61–3.03)
Coworker relationship	Unsatisfactory	19	27	1	1
	Satisfactory	204	159	1.82 (0.98–3.40)	1.01 (0.46–2.23)
Manager’s attitude towards time management	Poor attitude	85	83	1	1
	Good attitude	138	103	1.31 (0.88–1.94)	1.04 (0.62–1.75)

*COR* crude odd ratio, *CI* confidence interval, *AOR* adjusted odd ratio; 1: reference category.

*Significant at p < 0.05; **Significant at p < 0.001; n: sample size.

## Discussion

The present study's overall good work autonomy in public hospitals was 54.5% (95%CI: 54.48–54.53). It implies that those health professionals have above 50% work autonomy. However, healthcare practitioners need to have substantial work autonomy, which enables them to autonomously make decisions concerning patient care, treatment strategies, and other aspects of their professional responsibilities^36^.

According to this study; health professionals’ work autonomy in public hospitals could be impaired by organizational policy and strategy. Accordingly, the study revealed that health professionals in public hospitals who were satisfied with organizational policy and strategy were 2.34 times more likely to have good work autonomy than unsatisfied respondents. This finding was supported by the theory of Adam’s equity which stated that “if an employee is not fairly treated with organizational policy and strategy, the mistreatment could lead to undesired work autonomy and slow the pace of performance”^37^ This similarity could be justified due to the reason that health professionals who were satisfied with the policy and strategy of their organization might have a strong attachment to their institution. Therefore; such a kind of inclusive organizational policy and strategy could incorporate work autonomy into consideration and adjust situations to ensure their work autonomy.

Similarly, study participants who were satisfied with supervisor support were 7.20 times more likely to have good work autonomy as compared with their counterparts. This finding was in agreement with Herzberg’s motivation-hygiene theory which stated that “the more employees are motivated by supervisor support and other factors, the more they prevent dissatisfaction and such a motivation could encompass work autonomy”^38^. Therefore; if employees were satisfied with their supervisor’s support of their work, this supervisor support could be in terms of work autonomy in whatever type of health institutions they are being employed.

On the other hand, health service delivery planning practice was significantly associated with work autonomy. Accordingly, the likelihood of good work autonomy was 1.88 times higher among participants who had good health service delivery planning practices than their counterparts. This finding was in line with Pareto’s theory of time management, which stated that “there is no sufficient time to do each and everything, but there is time to do urgent and important things”. The central idea of this theory shows the prioritization of tasks; which is one of the pillars of planning^39^. If health professionals had a good plan, this would enable them to be autonomous in their work as a means of motivation provided by their managers for having a good plan.

Likewise, the odds of good work autonomy of anesthesia and radiology health professionals were 4.66 times more likely as compared to nurses. However, the work autonomy of pharmacy health professionals was decreased by 56% as compared to nurses. This could be due to differences in educational status among health professionals as the findings of this study indicated. It revealed that most anesthesia and radiology professionals hold a degree and above whereas most of the pharmacy professionals are diploma holders as compared to nurses. It might be also due to a nationwide shortage of anesthesia and radiology health professionals as compared to nurses. This might trigger employers to keep the autonomy of such insufficient professionals on top of the others to retain them in their institutions.

Moreover, marital status was significantly associated with work autonomy. Accordingly, being married was 4.26 times more likely to have good work autonomy as compared to unmarried respondents. This might be due to variation in the educational status of respondents as pointed out in this study. It showed that most of the married participants have a degree and above educational status as compared to unmarried groups. This might enable them to be highly aware of the value of work autonomy and they might strive to break any barriers to their autonomy. Furthermore, married health professionals might get social support from their spouses, which improves their psychological well-being and subsequently work autonomy^40,41^.

### Strengths and limitations of the study

Despite having typical limitations; this study tried to address extensively work autonomy by incorporating important variables that have been missed in previous studies; such as organizational policy, planning, supervisor support, living conditions, co-worker relationship, and working unit Besides, it was not separately studied work autonomy across specific health professional’s category which might affect a clearer picture of the relationship between work autonomy and profession.

The use of self-administered questionnaires may have some potential for reporting biases. Associated factors of work autonomy have been identified in this study. However, it would be more strong evidence if those factors were explored further using a qualitative study.

## Conclusions

The work autonomy in public hospitals of Northeast Ethiopia was low. Satisfaction with organizational policy and strategy, satisfaction with supervisor support, having good health service delivery planning practice, being married and type of profession were significantly associated factors in public hospitals.

This low work autonomy could affect the work engagement of health professionals and compromise the health service coverage and quality unless timely and appropriate interventions are taken. Policymakers need to emphasize their policy and strategy in the way to break the bottleneck of dissatisfaction of employees concerning supervisor support and organizational policy and strategy. Hospitals administrators should also encourage and train professionals to prepare their plans in their respective working units. Further study concerning work autonomy is needed to address the problem extensively by supporting a qualitative method in wider study settings.

## Data Availability

The results of this study were analyzed using the collected primary data. All the relevant data are included in this paper.

## Abbreviations

AOR: Adjusted odd ratio
CI: Confidence interval
IPH: Institute of Public Health
IRB: Institutional Review Board
SPSS: Statistical Package for Social Sciences
